# Prevalence of sarcopenia in women at stable weight phase after Roux-en-Y gastric bypass

**DOI:** 10.20945/2359-3997000000494

**Published:** 2022-06-02

**Authors:** Andreia Fabiana Bueno Buzza, Cristina Aquino Machado, Felipe Pontes, Letícia Guadanhim Sampaio, Júlia Soares Contador, Carolina Labigalini Sampaio, Rosana Bento Radominski, Cesar Luiz Boguszewski, Victoria Zeghbi Cochenski Borba

**Affiliations:** 1 Universidade Federal do Paraná Serviço de Endocrinologia e Metabologia do Hospital de Clínicas Departamento de Clínica Médica Curitiba PR Brasil Departamento de Clínica Médica, Serviço de Endocrinologia e Metabologia do Hospital de Clínicas da Universidade Federal do Paraná (SEMPR), Curitiba, PR, Brasil; 2 Universidade Federal do Paraná Faculdade de Medicina Centro de Ciências da Saúde Curitiba PR Brasil Centro de Ciências da Saúde da Faculdade de Medicina da Universidade Federal do Paraná, Curitiba, PR, Brasil; 3 Hospital Evangélico Mackenzie Curitiba PR Brasil Hospital Evangélico Mackenzie, Curitiba, PR, Brasil

**Keywords:** Obesity, bariatric surgery, sarcopenia, physical performance, strength

## Abstract

**Objective:**

Evaluating the prevalence of sarcopenia in women submitted to bariatric surgery – Roux-en-Y gastric bypass. Design: Observational, cross-sectional study.

**Subjects and methods:**

Women (18-65 years old) who underwent bariatric surgery (BG) ≥ 2 years and reached stable weight ≥ 6 months, were investigated. Control group (CG) comprised non-operated matched women with obesity. Body composition was determined through dual-energy X-ray absorptiometry. Low lean mass (LLM) was defined as appendicular lean mass index (ALM kg/height m^2^) < 5.5 kg/m^2^. Physical strength was assessed through dynamometer and sit-to-stand test (SST), whereas performance was assessed through 4-m gait speed and Short Physical Performance Battery Tests (SPPB). Sarcopenia was diagnosed in the presence of LLM and low strength.

**Results:**

One-hundred and twenty women (60 in each group, 50 ± 9.7 years old) were investigated. All anthropometric and body composition parameters were lower in BG than in CG, whereas strength and performance were similar between groups. Women with reduced strength presented high total fat mass and low physical activity level (p < 0.005). LLM was observed in 35% of BG and in 18.3% of CG (p = 0.04), whereas sarcopenia was diagnosed in 28.3% of BG and in 16.6% of CG (p = 0.12). Sarcopenic women in BG had better performance both in SST (p = 0.001) and SPPB (p = 0.004). Total lean mass (OR:1.41, 95% CI [1.18; 1.69], p < 0.001) and obesity (OR: 38.2 [2.27; 644.12], p < 0.001) were associated with sarcopenia in the multivariate analysis.

**Conclusion:**

Despite great weight loss, sarcopenia prevalence did not increase in BG and its presence was influenced by total lean mass and obesity.

## INTRODUCTION

Obesity is a worldwide epidemic of significant social and economic impact (1,2) that can lead to chronic diseases, functional limitations and higher mortality rates (2,3). Bariatric surgery is a therapeutic option when individuals’ lifestyle changes and medications are insufficient to achieve clinically significant body weight loss in patients with severe obesity (4,5); it is capable of promoting metabolic and cardiovascular improvements, as well as of reducing morbidity and mortality rates associated with obesity (6-8).

Overall, individuals with obesity have greater muscle mass and strength than individuals without obesity, since weight load and gravity act as stimuli to increase muscle formation (9,10). Nevertheless, weight loss is associated with lean mass reduction; thus, when people with obesity reach normal weight, this process may lead to decreased muscle strength (10). Muscle architecture and quality are influenced by reduced mobility, neural adaptations, slow muscle contractility, metabolic changes, and inflammation in individuals with obesity (9,10). Lean mass evaluation by dual-energy X-ray absorptiometry (DXA) lies on measuring non-bone and non-fat components of body composition; it is a component of sarcopenia diagnosis (11). Sarcopenia lies on low lean mass associated with decreased strength and/or functional capacity (11), which can significantly increase morbidity and mortality in comparison to LLM alone (12). Whenever sarcopenia is associated with obesity, it is called sarcopenic obesity. Sarcopenic obesity is featured by anomalous body composition, with higher fat mass proportion associated with sarcopenia (13,14), which leads to reduced physical activity and acts as vicious cycle to maintain this condition (15). According to estimates, sarcopenic obesity prevails in 2% of patients in the age group 60-69 years; however, its prevalence increases to 10% in patients older than 80 years (16); there are no data available about the statistics in younger individuals. Sarcopenic obesity prevalence rates in young populations remain unknown; they change depending on sarcopenia and obesity definitions and on the heterogeneity of the adopted diagnosis criteria and assessments. Moreover, most studies investigate older individuals, as seen in a recent systematic review (14) that do not present a standard definition of sarcopenic obesity (13,14). Prospective, cohort study conducted with 184 patients with obesity (79% women, mean age 42 years) subjected to pre-bariatric assessment has shown that fifteen (8%) women met the diagnostic criteria of sarcopenia (17). Bariatric surgery almost invariably predisposes patients to experience sarcopenic obesity onset, at least at the early rapid weight loss phase, due to significantly negative energy balance associated with this procedure (18). However, data on sarcopenia prevalence in the long-term after bariatric surgery, when patients reach stable weight, remain scarce and sparse in the literature. Thus, the aim of the current study was to investigate sarcopenia incidence during the post-bariatric surgery at stable-weight period in a group of women subjected to Roux-en-Y gastric bypass (RYGB), whose findings were compared to those of the control group, which comprised non-operated matched women with obesity.

## SUBJECTS AND METHODS

### Study design

Observational, cross-sectional study approved by the Ethics and Research Committee of our institution (Trial Registration number - Plataforma Brasil CAAE 51010815.6.0000.0096; 03/01/2016). Patients were invited to participate in the study for convenience during their routine visit in the Outpatient Obesity Clinic of our research center, from March 2017 to May 2019. All patients have signed the informed consent form to participate in the trial. Results were described by following the Strengthening the Reporting of Observational Studies in Epidemiology (STROBE) guidelines.

Inclusion criteria comprised women who underwent RGYB for at least 2 years, in the age group 18-65 years, who presented stable weight for at least 6 months and were under regular follow-up in our outpatient clinic. Women whose weight did not enable performing any exam included in the protocol, who were pregnant or in the postpartum period, who could not walk or used orthosis or prostheses, who had uncontrolled chronic diseases or took medications such as hormonal therapy, corticosteroids, immunomodulators, antiretrovirals and chemotherapy drugs or supplements known to affect body composition or bone metabolism were excluded from the study. Control group comprised non-operated women with obesity, who were followed-up in our outpatient clinic and did not want to undergo bariatric surgery; they were paired based on ethnicity and age, by following the same exclusion criteria.

Subgroup analysis has paired BG and CG – based on ethnicity, age and BMI – to avoid convenience sampling errors.

All patients and controls completed questionnaires focused on collecting socio-demographic data, information about current or pre-surgical comorbidities (diabetes, hypertension, thyroid disorders, sleep apnea), time of and age at menopause, hormone replacement therapy, alcohol intake and smoking habit, physical activity frequency and duration on a weekly basis (self-reported), medications and supplements in use, time of and age at surgery.

### Clinical and anthropometric measurements

Blood pressure and heart rate were measured before anthropometric assessments, based on standards set by the American Heart Association (AHA) (19). Weight was measured in calibrated scale after individuals took off their shoes and accessories; standard value of 0.5 kg (for clothing) was deducted from the measured weight. Height was measured with a stadiometer attached to the scale. Body Mass Index (BMI) was calculated as [weight (kg) divided by squared height in meters (m^2^) (20)]; total weight loss rate was calculated as [(initial weight – current weight) x 100/initial weight], and excessive weight loss rate (% EWL) was calculated as [(initial weight – current weight) x 100/initial weight – ideal weight (squared height multiplied by BMI of 25 kg/m^2^)] and total weight-loss rate (%TWL) was calculated as [(initial weight – current weight) x 100/initial weight]; it was effective if %EWL ≥ 50 and %TWL ≥ 25% (21). Waist circumference was measured in the largest abdominal perimeter between the last rib and the iliac crest; values ≤ 80 cm were considered normal (20). Neck circumference was measured at average neck height at the cricothyroid cartilage point; it was considered altered when ≥ 35 cm-36.5 cm was used as reference for correlation to obesity (22).

### Laboratory exams

Fasting blood samples were collected at the first visit. All laboratory tests were performed in the Clinical Chemistry Laboratory of the University Hospital for safety assessments such as fasting blood count, glucose, glycated hemoglobin, creatinine, albumin, lipid profile, liver enzymes, serum calcium, phosphorus, iron, vitamin B12, 25-hydroxyvitamin D (25OHD) and intact PTH assay.

### Body composition

Body composition was measured through dual-energy X-ray absorptiometry (DXA) in Hologic^®^ Horizon-A equipment (serial number 201383, Bedford, USA) by following guidelines set for daily calibration. Parameters used for body composition analysis comprised total lean mass in grams (TLM); appendicular lean mass (ALM), obtained by summing the lean mass of upper and lower limbs; total fat mass in kilograms (TFM); android fat in grams and percentage, and android/gynoid fat ratio.

### Physical strength

Upper limbs’ strength was evaluated through handgrip strength in Charder^®^ MG 4800 dynamometer. Patients remained in sitting position, with their feet resting on the floor, shoulders in adduction and neutral rotation, elbow flexed at 90°, neutral forearm and wrist in dorsiflexion position between 0°-30°. A demo was performed before measurements and positioning errors were corrected. The highest of three measurement values was used for classification purposes; values < 16 kg were considered low muscle strength (23). Lower limbs’ strength was measured based on the 5 times sit-to-stand test (SST), which was performed with patients sitting on a chair without armrest, without contact with the backrest, feet on the floor and aligned with the shoulders, arms crossed on the chest. The movement was performed by the examiner and patients were asked to repeat it for error correction purposes before the counting started. Subsequently, the time taken to perform the movement 5 times in a row was recorded in seconds; patients who needed > 15 seconds to do it were categorized as having muscle weakness (23).

### Physical performance

Physical performance was assessed through gait speed and Short Physical Performance Battery Tests (SPPB) by following the National Institutes of Health's (NIH) protocols recommended by EWSOP-2 (23). The Portuguese-validated SPPB was applied (24); it comprises three tests, namely: gait-speed, balance (side-by-side, semi-tandem and full-tandem) and SST. Test scores were specific to each SPPB protocol stage; the total sum of tests ≤ 8 points was considered low physical performance. With respect to the gait-speed test, women were asked to walk 4 meters in normal speed; this distance was marked by tapes fixed on the floor – walking time scored 0 if participants were unable to complete the test; score 1 corresponded to walking time > 8.7 seconds; score 2, to walking time ranging from 6.21 to 8.7 seconds; score 3, from 4.82 to 6.2 seconds; and score 4, to walking time < 4.82 seconds. Based on the balance test, participants should be able to remain in each of three test positions for 10 seconds, namely: standing with feet side by side, semi-tandem (standing with one foot partially forward) and full-tandem (standing with one foot fully forward). Patients scored 1 point when they remained in the first two positions for 10 seconds; if they failed to do so, they scored 0. Participants able to remain in the third position for 10 seconds scored 2 points, those who held the position for 3 to 9.99 seconds scored 1 point, and those who held it for < 3 seconds, or were unable to do so, scored 0 point (23,24). Gait speed in meter per seconds (m/s) was used to calculate the walking speed recorded in SPPB; it was considered low when ≤ 0.8 m/s (23,24).

### Low lean mass and sarcopenia definition

Sarcopenia diagnosis was presumed in all patients. Low lean mass (LLM) was defined as ALM index < 5.5 kg/m^2^, which was calculated through ALM/h^2^, based on EWSOP-2 (23). Sarcopenia was diagnosed in the presence of LLM associated with low physical strength; severe sarcopenia was diagnosed when physical performance also decreased (23).

### Statistical analysis

Statistical analysis was performed in Stata/SE software v.14.1. (Stata Corp LP – USA). Sample normality was checked based on Jarque-Bera test. Data were expressed as absolute and relative frequencies for qualitative variables, and as mean ± standard deviation (SD) or median (minimum and maximum) for quantitative variables. Student's t or non-parametric Mann-Whitney test were used to compare two variables, whereas analysis of variance (ANOVA) was used to compare multiple variables. Chi-Square, Fisher's Exact and Binomial tests were used to assess the association of qualitative variables. Pearson and Spearman's correlation coefficients were estimated to investigate correlation between quantitative variables. The influence of variables of interest on the likelihood of identifying pre-sarcopenia and sarcopenia was assessed through Logistic Regression. P-values < 0.05 indicated statistically significant.

## RESULTS

Two hundred and twenty women with obesity followed-up at our clinic were invited to participate in the study, which included both non-operated and post-bariatric patients. One hundred and thirty-one of them accepted the invitation and signed the informed consent form, but 11 patients were excluded due to chronic liver disease, active malignancy, use of interfering medications or simply dropped out. The final sample comprised 120 women: 60 in the post-bariatric group (BG) and 60 in the control group (CG), at mean age 50 ± 9.7 years, roughly two-thirds were Caucasian. Both groups were similar in menopause status, alcohol intake and smoking habit. %TWL and %EWL in BG were 35.8 ± 8.7% (sarcopenic (S-BG): 31 ± 6.1% *vs.* non-sarcopenic (NS-BG): 37 ± 8.8%, p = 0.003) and 78.6 ± 20.0% (S-BG: 71 ± 15% *vs.* NS-BG: 81 ± 20%, p = 0.06), respectively. As expected, weight, BMI, as well as waist and neck circumference were significantly higher in CG than in BG (Table 1). There was no correlation between %EWL and parameters such as body composition, physical strength, and performance. Hypertension was more prevalent in CG (p = 0.001). BG exercised more, both in frequency and duration, as well as consumed more supplements than CG (p < 0.005 for all; Table 1). Women in BG who did not exercise on a regular basis took longer to perform SST than the most active ones (p < 0.001). %TFM was inversely correlated to physical activity in both groups (minutes/week) (r = - 0.45; p = 0.001).

**Table 1:** Clinical and demographic features of the post-bariatric (BG) and control groups (CG)

Features			BG (N = 60)	CG (N = 60)	P value
Age (years)			50.3 ± 9,7	50.2 ± 9.7	0.948
Weight (kg)			75.1 ± 5.1	87.8 ± 5.5	<0.001
Body mass index (kg/m^2^)			30.2 ± 4.8	35.5 ± 5.6	0.001
Waist circumference (cm)			98.9 ± 13.3	108.6 ± 18.6	0.001
Neck circumference (cm)			35.7 ± 2.7	38.5 ± 3.5	<0.001
Menopause (years)					
	Time		12.2 ± 9.4	9.3 ± 7.0	0.159
	Age at onset		46.2 ± 6.4	48.3 ± 5.5	0.161
Comorbidities					
	Diabetes with insulin		12 (20%)	9 (15%)	0.471
	Diabetes without insulin		1 (1.7%)	3 (5.0%)	0.309
	Hypertension		16 (27.1%)	34 (56.7%)	0.001
	Hypothyroidism		34 (16.7%)	11 (18.3%)	0.810
Lifestyle habits					
	Alcohol intake		4 (6.7%)	1 (1.7%)	0.171
	Smoking habit		3 (5.0%)	5 (8.3%)	0.464
	Physical activity		37	26	
		frequency (day/week)	4.4 ± 2.0	3.3 ± 1.6	0.032
		duration (min/week)	266.6 ± 163.7	46.9 ± 12.7	<0.001
Supplements					
	Calcium		54 (90%)	3 (5%)	<0.001
	Vitamin D		51 (86%)	1 (2%)	<0.001
	Vitamin B12		51 (85%)	0	<0.001
	Multivitamins		56 (95%)	1 (2%)	<0.001

Serum iron, fasting glucose, glycated hemoglobin, creatinine, 25OHD, phosphorus, liver enzymes and blood count levels did not differ between groups. BG showed albumin, serum calcium corrected by albumin, hemoglobin, LDL-cholesterol, total cholesterol and triglycerides lower than those of CG. On the other hand, BG presented HDL-cholesterol, PTH and vitamin B12 values higher than those of CG. There was not difference in the number of patients diagnosed with secondary hyperparathyroidism between the two groups.

All body composition parameters were significantly lower in BG than in CG (Table 2). LLM was observed in 21 (35%) women in BG and in 11 (18.3%) women in CG (p = 0.04). Hand-grip strength was similar between groups; low strength was observed in 4 (6.7%) women in BG and in 3 (5%) women in CG. There was higher TFM, both in grams (p = 0.01) and percentage (p = 0.02), in women with reduced hand-grip strength than in those with normal strength.

**Table 2:** Body composition parameters in the post-bariatric (BG) and control groups (CG) assessed through dual-energy X-ray absorptiometry (DXA)

Body composition parameters	Groups		p value
	**BG (N = 60)**	**CG (N = 60)**	
Total Lean Mass (g)	38,913 ± 6.0	43,006 ± 6.1	<0.001
Total Fat Mass (%)	43.7 ± 4.7	47.5 ± 3.9	<0.001
Android fat (g)	2,387 ± 978	3,528 ± 1.11	<0.001
Android fat (%)	39.5 ± 7.2	47.4 ± 4.7	<0.001
Android/gynoid fat	0.9 ± 0.1	1.0 ± 0.1	<0.001
Appendicular Lean Mass (kg)	16.9 ± 3.3	18.6 ± 3.0	<0.001
Appendicular Lean Mass index (ALM/height^2^) (kg/m^2^)	6.88 ± 1.0	7.58 ± 1.1	<0.001
	**S-BG (N = 17)**	**S-CG (N = 10)**	
Total Lean Mass (g)	35,036 ± 4,528	35,175 ± 2,674	0.930
Total Fat Mass (g)	2,8591 ± 7045	32,796 ± 6,100	0.140
Total Fat Mass (%)	44 ± 5.4	47 ± 4.6	0.110
Android fat (g)	2,011 ± 908	2,758 ± 881	0.050
Android fat (%)	38 ± 7.9	47 ± 7.1	0.010
Android/gynoid fat	0.85 ± 0.16	0.98 ± 0.17	0.050
Appendicular Lean Mass (kg)	18.9 ± 2.5	18.3 ± 2.6	0.590
Appendicular Lean Mass index (ALM/height^2^) (kg/m^2^)	5.42 ± 0.1	5.42 ± 0.1	1.000

S-BG: bariatric group with sarcopenia; NS-BG: bariatric group without sarcopenia; S-GC: control group with sarcopenia; NS-CG: control group without sarcopenia.

**Figure 1:**
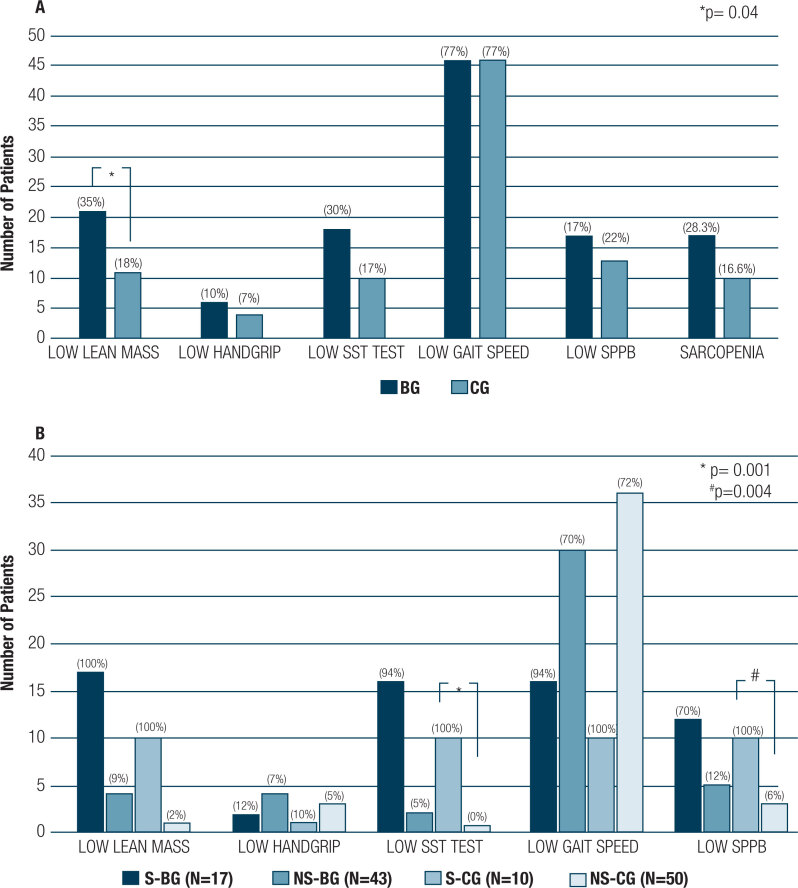
Number of patients with sarcopenia parameters in the bariatric and control groups (**A**) and in bariatric and control groups based on sarcopenia diagnosis (**B**). NS-BG, post-bariatric group without sarcopenia; S-BG, post-bariatric group with sarcopenia; NS-CG, control group without sarcopenia; S-CG, control group with sarcopenia.

Sarcopenia was diagnosed in 17 (28.3%) women in BG and in 10 (16.6%) women in CG (p = 0.12). Sarcopenic and non-sarcopenic patients in BG (p = 0.68) and CG (p = 0.08) presented similar mean age. Comorbidities did not show significant difference before and after bariatric surgery. The whole group of women with sarcopenia presented waist circumference (96 ± 9.9 cm *vs.* 107 ± 14.8 cm, p = 0.002) and BMI (30.8 ± 3.1 kg/m^2^ *vs.* 33.9 ± 6.3 kg/m^2^, p = 0.001) lower than those of the non-sarcopenic group; however, sarcopenic and non-sarcopenic BMI in BG (p = 0.75) and CG (p = 0.04) presented similar results. There was not difference in other body composition parameters between BG and CG, except for lean mass, which was a component of sarcopenia diagnosis. Among 27 patients with sarcopenia, only 2 (7.4%) in BG and 1 in CG (p = 0.60) have shown reduced handgrip strength. SST was the main strength criterion used to diagnose sarcopenia in both groups. Sarcopenic women in both groups presented similar lean mass, whereas sarcopenic women in CG had the worst performance in SST (p = 0.001) and SPPB (p = 0.004) (Table 3, Figure 1). Overall, gait speed was low in 46 patients; there was not difference in the prevalence of low gait speed between the investigated groups, and between sarcopenic women in BG and CG. SPPB score ≤ 8 was observed in 17 and 13 women in BG and CG, respectively. Low SPPB was observed in 12 patients in BG, as well as in 10 patients in CG (Table 3). The subgroup analysis of patients (35 women in BG and 35 women in CG) matched by age, ethnicity and BMI kept the same results, which confirmed that CG had more patients with longer SST than BG, p = 0.001 (Table 4). Supplementary Table 1 shows the comparison of all sarcopenia components between the total and paired groups.

**Table 3:** Comparison of diagnostic sarcopenia parameters in post-bariatric women without (NS-BG) and with sarcopenia (S-BG) to those recorded for women in the control group without (NS-CG) and with sarcopenia (S-CG)

Variables	NS-BG (N = 43)	S-BG (N = 17)	NS-CG (N = 50)	S-CG (N = 10)	P*
LM index (ALM/h^2^)	7.2 ± 1.0	5.42 ± 0.15	7.8 ± 1.0	5.42 ± 0.10	1.000
LLM, n (%)	4 (9)	17 (100)	1 (2)	10 (100)	1.000
Handgrip strength (kg)	28 ± 6.1	21 ± 6.6	26 ± 6.9	23 ± 5.5	0.300
Low handgrip strength (%)	4 (9)	2 (12)	3 (6)	1 (10)	0.600
SST (s)	11 ± 1.7	15.4 ± 1.8	12 ± 0.6	18.10 ± 1.4	0.001
SST > 15s n (%)	2 (5)	16 (94)	0	10 (100)	0.430
GS (m/s)	0.8 ± 0.20	0.62 ± 0.14	0.76 ± 0.12	0.60 ± 0.09	0.700
Low GS, n (%)	30 (70)	16 (94)	36 (72)	10 (100)	0.430
SPPB score	10 ± 1.8	8.1 ± 1.6	10 ± 0.8	6.3 ± 1.0	0.004
Low SPPB, n (%)	5 (12)	12 (70)	3 (6)	10 (100)	0.060

LM: lean mass; LLM: low lean mass; ALM/h^2^: appendicular lean mass divided by squared height; SST: sit-to-stand test; GS: gait-speed; SPPB: Short Physical Performance Battery tests; s: seconds; m: meters.

* p value comparing S-BG vs S-CG.

**Table 4:** Clinical and demographic features of the post-bariatric (BG) and control groups (CG) paired by age, ethnicity, and body mass index (BMI)

Characteristics			BG (N = 35) N (%)	CG (N = 35) N (%)	P value
Age (years)			50.5 ± 8.4	50.6 ± 9.0	0.94
Weight (kg)			78.6 ± 9.4	80.5 ± 5.5	0.43
BMI (kg/m^2^)			31.6 ± 3.6	32.0 ± 3.7	0.63
Waist circumference (cm)			101 ± 12	104 ± 10	0.35
Neck circumference (cm)			36.5 ± 2.7	37.2 ± 3.5	0.29
Menopause (years)					
	Time		11.5 ± 9.0	8.3 ± 7.0	0.28
	Age at onset		47 ± 5.4	49 ± 5.0	0.21
Comorbidities					
	Diabetes with insulin		1 (3)	2 (6)	0.55
	Diabetes without insulin		5 (14)	4 (11)	0.72
	Hypertension		13 (37)	12 (34)	0.80
	Hypothyroidism		7 (20)	9 (26)	0.5
Lifestyle habits					
	Alcohol intake		3 (9)	1 (3)	0.55
	Smoking habits		2 (6)	3 (9)	0.30
	Physical activity		19 (54)	16 (46)	0.47
		duration (min/week)	277 ± 171	47 ± 14	<0.001
Supplements					
	Calcium		31 (89)	2 (6)	<0.001
	Vitamin D		29 (83)	1 (3)	<0.001
	Vitamin B12		29 (83)	0	<0.001
	Multivitamins		32 (95)	0 (2)	<0.001
LLM index			5.3 ± 0.4	5.5 ± 0.35	0.79
LLM			17 (49)	10 (29)	0.10
Low HGS			3 (9)	1 (3)	0.30
Low GS			27 (77)	26 (74)	0.78
SST>15s			13 (37)	10 (29)	0.45
Sarcopenia			13 (37)	10 (29)	0.45
Severe sarcopenia			5 (14)	4 (11)	0.72

LLM: low lean mass; SST: sit-to-stand test; GS: gait-speed; SPPB: Short Physical Performance Battery tests; s: seconds; m: meters.

* p value.

Time elapsed since surgery was similar between sarcopenic (S-BG) and non-sarcopenic (NS-BG) women (6.0 ± 3.8 and 6.6 ± 3.9 years, respectively), and it was not correlated to sarcopenia diagnosis. On the other hand, %TWL was significantly higher in NS-BG (39.8 ± 8.6% in NS-BG *vs.* 34.3 ± 8.3% in S-BG, p = 0.01) and %EWL has shown trend to higher values (81 ± 20% in NS-BG *vs.* 70 ± 15% in S-BG, p = 0.05). Multivariate analysis considered sarcopenia diagnosis as dependent variable controlled by all significant variables in the univariate analysis (BMI, waist circumference, TLM, albumin, and age); results have shown that low TLM (OR: 1.41, 95% CI [1.18; 1.69], p < 0.001) and obesity (OR: 38.2 [2.27; 644.12], p < 0.0012) were the most significant risk factors.

## DISCUSSION

The current observational cross-sectional study has shown sarcopenia diagnosis in 28% of women subjected to RYGB surgery, at least 2 years after they reached stable weight. This prevalence was not significantly different from that (nearly 17%) observed in the control group, which comprised non-operated women with obesity, despite the striking differences in body composition between groups, such as significant difference in LLM prevalence, which is one of sarcopenia's components. In addition, sarcopenic women in both investigated groups presented similar TLM, although sarcopenic patients in BG have shown better strength and performance parameters (SST and SPPB scores).

Sarcopenia definition can change from study to study (11). Changes in cut-off points or methods to measure muscle mass, strength or performance are often observed in different guidelines (11,25). The current study has used the most recent EWGSOP-2 criteria, which take into consideration both LLM (ALM index < 5.5 kg/m^2^) and low strength for sarcopenia diagnosis, as well as categorizes patients as having severe sarcopenia when their physical performance is also compromised (23).

The literature has few studies focused on investigating sarcopenia in post-bariatric patients. A prospective study conducted with 19 patients followed-up during 24 months after surgery did not observe sarcopenia in any patient (26). Some authors have claimed that handgrip strength could be used alone to differentiate patients with sarcopenia, since it is considered an independent muscle mass predictor and shows good correlation to functional capacity in elderly individuals (27). However, only 6% of women assessed in the current study had low handgrip strength, and it suggested that this parameter is not suitable for sarcopenia diagnosis after bariatric procedures. On the other hand, SST was the best individual parameter used to identify sarcopenia in our series; thus, further studies should be conducted to establish its diagnostic value in patients with obesity and in post-bariatric patients. Nevertheless, as observed in older adults, multiple tests used to evaluate performance and strength are better sarcopenia predictors than any single test (27,28).

The correct identification of sarcopenia in bariatric patients is an important factor to help assessing muscle quantity and quality, since muscle strength is one of the best predictors of health and mortality outcomes, regardless of muscle mass (23). Patients with severe obesity, who were subjected to bariatric procedures, have experienced fast and significant weight loss associated with nutritional disorders that can potentially increase the risk of sarcopenia (29). In fact, LLM diagnosis was significantly higher in BG in the current study, a fact that confirmed important muscle loss after surgery, although it was not followed by substantial decrease in muscle strength, as seen in the non-significant difference in sarcopenia between BG and CG. These findings were substantiated by the paired sub-analysis of BG and CG, which showed that although the amount of lean muscle mass decreased in BG, the strength was similar to that of non-operated patients with the same BMI. This outcome resulted in similar sarcopenia prevalence, which was not expected after a bariatric surgery.

In fact, obesity *per se* could contribute more than sarcopenia to lower physical capacity (30) since adiposity is a stronger physical function predictor (31). Accordingly, women in BG recorded better physical activity, SST and SPPB scores than those in CG.

The %EWL observed in the present study was higher than that of other series available in the literature (32), but it was not correlated to LLM or to sarcopenia diagnosis. One possible explanation for this outcome is that changes in fat mass and lean body mass after bariatric surgery are not linear. Studies have shown that decrease in total lean mass is more expressive in the acute phase after bariatric surgery, whereas fat mass loss is more expressive over the years (33). Patients in this study were investigated during the post-surgical phase at stable weight. In addition, there was inverse correlation between fat amount and strength in the whole group of patients.; this outcome may be explained by fat infiltration in patients’ muscles, a phenomenon that cannot be detected in DXA (34). Another possible explanation for it was shown in a metanalysis, according to which, exercise training in bariatric patients was not associated with changes in lean body mass, although it was effective in optimizing weight and fat mass loss, as well as in improving physical fitness, since BG was more physically active than CG (35).

TLM and obesity were associated with sarcopenia in the herein conducted multivariate analysis, whereas age, time since surgery and weight loss amount did not influence the diagnosis. Women in BG were slightly older than the mean age of post-bariatric subjects, but they were still not in the age range most often associated with sarcopenia (35). Sarcopenia does not appear to be correlated to BMI in individuals older than 65 years, since different studies have shown higher prevalence of it in patients with, and without, obesity (30).

The cross-sectional nature of the current study was one of its main limitations, due to lack of pre-surgical assessment of patients’ body composition, physical strength, and performance to enable evaluating and comparing BG's results. It is hard to find an ideal control group for post-bariatric patients with similar body composition; although a subanalysis of matched groups was performed, the difference in body composition between groups could have affected the comparison of sarcopenia prevalence. It only included adult women because they are more often subjected to bariatric surgery in our institution, and because they are at higher risk of developing sarcopenic obesity (32-34). The current results cannot be extrapolated to men, mainly due to gender-related differences in body composition and strength, which are the factors defining sarcopenia. Likewise, this study has only investigated patients subjected to RYGB, and it should not be extrapolated to other surgical techniques. Despite the sample size limitation, the current results provided novel information and opened new perspectives in a field with significantly scarce publications.

Finally, women subjected to RYGB did not show increased sarcopenia prevalence in comparison to non-operated women with obesity, despite the significant weight loss, higher LLM frequency and striking differences in body composition. Women in BG have shown better physical performance among those diagnosed with sarcopenia.

## Supplementary Table 1 Comparison of sarcopenia diagnostic parameters in total and matched (age, ethnicity, and BMI) post-bariatric women without (NS-BG) and with sarcopenia (S-BG) and in non-operated obese women without (NS-CG) and with sarcopenia (S-CG)

**Table:**

Variables		NS-BG Matched: 22 Total: 43	S-BG Matched: 13 Total: 17	NS-CG Matched: 25 Total: 50	S-CG Matched: 10 Total: 10	P value*
LM index (ALM/h^2^)	Matched	7.4 ± 1.0	5.42 ± 0.1	7.28 ± 1.0	5.41 ± 0.2	1.00
	Total	7.2 ± 1.0	5.42 ± 0.15	7.8 ± 1.0	5.41 ± 0.10	1.00
LLM, n (%)	Matched	4 (18)	13 (100)	0	10 (100)	1.00
	Total	4 (9)	17 (100)	1 (2)	10 (100)	1.00
Handgrip strenght (kg)	Matched	28 ± 6,5	20.7 ± 7.5	27.6 ± 6.5	23.7 ± 5.5	0.31
	Total	28 ± 6.1	21 ± 6.6	26 ± 6.9	23.7 ± 5.5	0.30
Low handgrip strenght n (%)	Matched	1 (5)	2 (15)	0	1(10)	0.93
n (%)	Total	4 (9)	2 (12)	3 (6)	1 (10)	0.89
SST (s)	Matched	11.5 ± 4.8	14.5 ± 4.5	11.1 ± 1.5	18.10 ± 1.4	0.001
	Total	11 ± 1.7	15.4 ± 1.8	12 ± 0.6	18.10 ± 1.4	0.001
SST>15 s, n (%)	Matched	1 (5)	12 (92)	0	10 (100)	0.67
	Total	2 (5)	16 (94)	0	10 (100)	0.74
GS (m/s)	Matched	0.7 ± 0.17	0.62 ± 0.20	0.80 ± 0.12	0.60 ± 0.10	0.72
	Total	0.8 ± 0.20	0.62 ± 0.14	0.76 ± 0.12	0.60 ± 0.10	0.70
Low GS, n (%)	Matched	15 (68)	12 (92)	16 (64)	10 (100)	0.67
	Total	30 (70)	16 (94)	36 (72)	10 (100)	0.74
SPPB score	Matched	10 ± 1.0	6.6 ± 1.0	9.9 ± 1.0	6.3 ± 1.0	0.50
	Total	10 ± 1.8	8.1 ± 1.6	10 ± 0.8	6.3 ± 1.0	0.004
Low SPPB, n (%)	Matched	3 (14)	9 (69)	2 (8)	10 (100)	0.16
	Total	5 (12)	12 (70)	3 (6)	10 (100)	0.06

LM index: lean mass index; LLM: low lean mass; ALM/h^2^: appendicular lean mass divided by squared height; SST: sit-to-stand test; GS: gait-speed; SPPB: Short Physical Performance Battery tests; s: seconds; m: meters.

* p value comparing S-BG vs. S-CG. Total (BG:60, CG:60): total analyzed patients. Matched (BG:35, CG:35): patients paired by age, ethnicity and BMI.
